# Role of Serum Uric Acid as a Protective Biomarker in Patients with Different Histopathological Grades of Oral Squamous Cell Carcinoma: a Case-Control Study

**DOI:** 10.1155/2020/5185423

**Published:** 2020-09-12

**Authors:** Soheila Manifar, Arezoo Rahimzamani, Mohammad Shirkhoda, Mohammad Nikkhah Ghamsari, Mahin Bakhshi

**Affiliations:** ^1^Department of Oral Medicine, School of Dentistry, Tehran University of Medical Sciences, Imam Khomeini Hospital Complex, Tehran, Iran; ^2^Department of Oral Medicine, School of Dentistry, Shahid Beheshti University of Medical Sciences, Tehran, Iran; ^3^Department of Surgery, Cancer Institute, Tehran University of Medical Sciences, Tehran, Iran; ^4^Dentist, School of Dentistry, Shahid Beheshti University of Medical Sciences, Tehran, Iran

## Abstract

The antioxidant properties of uric acid may have a protective effect against the formation of oxygen radicals and subsequently against carcinogenicity. The present study aimed at evaluating the serum level of uric acid in patients with oral squamous cell carcinoma (OSCC) with different histopathological grades. In this descriptive-analytical, case-control study, patients with OSCC and healthy controls were selected and matched regarding age and gender. The serum samples were collected from patients (before radiotherapy and chemotherapy) and controls, and their serum uric acid levels were measured enzymatically. Data were analyzed using independent *t*-test and ANOVA at 0.05 level of significance. The mean serum uric acid level in OSCC patients (4.2 ± 1.5 mg/dL) and healthy controls (4.38 ± 1.22 mg/dL) was not significantly different (*P* = 0.377). No significant association was noted between the histopathological grade of OSCC and mean serum uric acid (*P* = 0.781). The serum uric acid level had a direct significant correlation with age in OSCC patients (*P* < 0.001). The results of this study did not support the role of uric acid as a protective biomarker in OSCC. Further prospective studies are recommended to better elucidate the role of uric acid in the etiology of OSCC.

## 1. Introduction

In recent years, cancer has been one of the leading causes of mortality in human societies. It is the second most common cause of death after cardiovascular diseases in the United States [1, 2] and the third most common cause of death in Iran [3].

Oral cancer is among the most common cancers worldwide and is a public health dilemma. According to histopathological examinations, squamous cells are the origin of nearly 90% of oral cancers, referred to as oral squamous cell carcinoma (OSCC) [4].

Oxidative stress is one of the indicators of cancer development, resulting from an imbalance between the levels of oxidative and antioxidant agents in biological systems. Many of the oxidative stress biomarkers produced by oxygen free radicals are involved in the pathogenesis of many diseases, including cancer [5].

Uric acid is the final enzymatic product of the breakdown of purine nucleotides and free bases in the human body [6]. According to some previous studies, a reduction in serum uric acid level has been associated with an increase in the risk of lung cancer, oral cancer, and laryngeal cancer [7–10]. However, some others have reported that uric acid plays a role as a prooxidant under conditions of oxidative stress through reactions with nitric oxide and impairs the vascular epithelial function and leads to the occurrence of systemic diseases [9–14].

Uric acid appears to elicit an antioxidant defense mechanism against oxidative stress and the aging processes caused by free radicals that are also associated with DNA damage, attachment, migration, proliferation, and regulation of tumor cells, and mortality. On the other hand, the degradation of tumor cells may increase the serum uric acid level, which stimulates the immune system (CD8^+^ T-lymphocytes in particular) and enhances the defense mechanisms against cancer by inducing cytotoxic cell death and inhibiting the proliferation and migration of tumor cells. These findings support the positive association of serum uric acid level and survival in patients with colon cancer and nasopharyngeal carcinoma [15]. In contrast, elevated serum uric acid levels are inversely correlated with low adiponectin levels, and low adiponectin levels may overactivate the phosphorylation of PI3K/Akt (phosphoinositide 3-kinase/B kinase protein) and signaling pathways and eventually lead to an increase in the proliferation of tumor cells [13]. Given the paradoxical role of uric acid as an antioxidant and prooxidant in the occurrence of OSCC, the aim of the present study was to evaluate the serum uric acid level in a group of patients with OSCC.

## 2. Materials and Methods

### 2.1. Primary Outcome Measures

The main objectives of this study were (I) to compare the mean serum level of uric acid between OSCC patients and healthy controls and (II) to compare the mean serum level of uric acid in OSCC patients with different histopathological grades.

### 2.2. Study Design

This descriptive-analytical, case-control study was performed on newly diagnosed patients with OSCC, confirmed by a pathologist at Imam Khomeini Cancer Institute in Tehran. The patients were selected by convenience sampling.

### 2.3. Participants

Forty newly diagnosed patients with OSCC were selected. The patients had definite histopathological diagnosis of OSCC according to their pathology report. The inclusion criterion for OSCC patients was (I) patients whose diagnosis of OSCC had been confirmed histopathologically. The exclusion criteria were (I) initiation of treatment (radiotherapy and/or chemotherapy), (II) systemic diseases such as rheumatoid arthritis, type II diabetes, obesity, hypertension, cardiovascular diseases, renal failure, and gout due to decreased level of nitric oxide and vascular epithelial malfunction as a result of high level of uric acid [14, 16].

Healthy controls were selected among healthy individuals who had presented for regular check-up and had no systemic conditions. The control group matched the patient group in terms of age and gender.

### 2.4. Data Collection

The patients' information was recorded in a checklist anonymously and included gender, age, histopathological grade of tumor, location of tumoral lesion, and uric acid level.

For OSCC patients, the histopathological grade of OSCC was extracted from their pathology report. The classification system used for grading was as follows [17]:

G1: Well differentiated (low grade)

G2: Moderately differentiated (intermediate grade)

G3: Poorly differentiated (high grade)

G4: Undifferentiated (high grade)

After obtaining written informed consent from the participants, blood samples (5 cc) were collected from the radial vein of patients and healthy controls during 8-10 a.m. The serum level of uric acid was measured using a uric acid assay kit (Bionik, Germany), which enzymatically measures the serum uric acid at 520 nm wavelength. According to the information provided in the kit, the normal mean serum level of uric acid is 3.5-7.2 mg/dL in males and 2-6 mg/dL in females.

### 2.5. Statistical Analyses

The minimum sample size was calculated to be 37 in each group according to a previous study by Ara et al. [18], assuming 1.5-unit difference between the two groups, alpha = 0.05, study power of 0.8, and standard deviation of 2.3.

Data were analyzed using SPSS version 22 (SPSS Inc., IL, USA). The Kolmogorov-Smirnov test was used to evaluate the distribution of data. Independent *t*-test, ANOVA, and Pearson's correlation coefficient were used for data analysis. The level of significance was set at 0.05.

We used a 95% confidence interval for this study. In other words, we predicted a 5% probability of errors in the results. No bias was found in this study.

## 3. Results

### 3.1. Characteristics of the Participants

Of a total of 80 participants, 40 were males (*n* = 20 in each group) and 40 were females (*n* = 20 in each group) (*P* = 1).

The mean age of patients was 46.86 ± 9.66 years (range 26-66 years), and the mean age of healthy controls was 47.3 ± 8.76 years (range 31-66 years). According to the Kolmogorov-Smirnov test, data regarding the age of the two groups were normally distributed. Thus, the student *t*-test was applied to compare the two groups regarding age, which revealed no significant difference (*P* = 0.2).

The frequency of lesions according to their location in the oral cavity was also studied. The tongue was the most common location for the occurrence of OSCC.

Of 40 patients who participated in this study, the grade of OSCC was mild in 18 (22.5%), moderate in 14 (17.5%), and high in 8 (10%) patients. We did not have any grade 4 cases.

Table 1 presents the gender and age of patients, location of OSCC lesions, and histopathological tumor grade.  Table 2 shows the frequency of OSCC lesions in different locations in the oral cavity according to their histopathological grade.

### 3.2. Assessment of the Serum Uric Acid Level

Table 3 shows the mean serum uric acid level in the patient and control groups. The Kolmogorov-Smirnov test confirmed the normal distribution of serum uric acid level data in the patient and control groups (*P* = 0.2). Thus, *t*-test was used to compare the mean serum uric acid level between the two groups, which revealed.no statistically significant difference in this respect (*P* = 0.377).

Table 4 shows the mean serum level of uric acid in male and female patients. The Kolmogorov-Smirnov test confirmed the normal distribution of serum uric acid data in male and female patients. Thus, *t*-test was used to compare the mean serum uric acid level between males and females, which showed no significant difference (*P* = 0.201).

Since the data regarding age and serum uric acid level were normally distributed, the Pearson's correlation coefficient was applied to assess the correlation between these two variables, which showed a direct significant correlation between age and serum uric acid level (*r* = 0.714, *P* < 0.0001).

Assessment of the relationship of uric acid level and histopathological grade revealed that the mean serum level of uric acid in patients with mild grade of OSCC was 4 ± 1.36 mg/dL, while this value was 4.35 ± 1.83 and 1.39 ± 4.36 in patients with moderate and high grades of OSCC, respectively. ANOVA was applied to compare the serum uric acid level between the three histopathological grades, which showed no statistically significant difference in serum level of uric acid between the three groups (*P* = 0.781, Table 5).

## 4. Discussion

Uric acid is the final product of the purine metabolism in the human body [6]. Also, some studies have shown that uric acid can eliminate the free radicals as an essential antioxidant in the human plasma [7, 19–22]. About half of the antioxidant capacity of the human plasma is attributed to the presence of uric acid [20]. Up to 50% of the serum uric acid is believed to originate from the food sources. Serum uric acid level is also affected by alcohol consumption, use of fructose-containing sugars, deficiency in purine metabolism, renal dysfunction, hyperinsulinemia, intake of medications such as diuretics, and genetic factors [23, 24].

This study is aimed at determining the serum level of uric acid in patients with different histopathological grades of OSCC to find possible associations. The serum uric acid was not significantly different in OSCC patients and healthy controls (*P* = 0.377). This finding was similar to the results of a prospective cohort study by Hiatt and Fireman [25], and another study by Kolonel et al. [16] that did not find any association between the serum level of uric acid and cancer incidence. Also, Almadori et al. [26] reported that the salivary concentration of uric acid in patients with head and neck squamous cell carcinoma was not significantly different from that in the healthy control group. This may confirm the parallel relationship between the salivary and serum levels of biomarkers, because, in many studies, saliva has been used as a diagnostic tool for the detection of systemic and oral conditions [4, 23, 27, 28].

However, some other studies found different results about the serum and salivary uric acid levels in patients with head and neck squamous cell carcinoma. Lawal et al. [23] reported that serum uric acid concentration in patients with head and neck squamous cell carcinoma was significantly lower than that in healthy controls. Some others also reported lower salivary uric acid levels in patients with oral cancer [15, 24, 28–30]. In another study in Austria, Strasak et al. [29] found that higher serum level of uric acid was associated with a higher risk of mortality, although they did not observe a correlation between the serum uric acid level and cancer incidence.

Yiu et al. [31] evaluated the serum uric acid level and subsequent development of cancer in cancer-free individuals. They found that altered uric acid levels were associated with the overall and specific risk of some cancer types, including the colorectal, hepatobiliary, kidney, and nonmelanoma skin cancer in males, and head and neck, and other cancer types in females. However, an inverse correlation was noted for pulmonary and central nervous system cancers in males and breast, lymphatic, hematological, and central nervous system malignancies in females.

Lea [32] in an observational analytical study in Indonesia found a strong correlation between the serum uric acid levels and stages of malignancy in gastric cancer patients. They concluded that any increment in uric acid levels would be followed by an increase in stages of malignancy in gastric cancer. Yuan et al. [30] suggested that serum uric acid level could be a good marker for the evaluation of tumor metastasis in patients with rectal cancer. They found that serum uric acid concentration was correlated with the presence of C-reactive protein and carcinoembryonic antigen in patients with rectal cancer and the presence of an inflammatory response. In fact, uric acid has been shown to increase inflammation and oxidative stress, which can enhance tumor cell proliferation and angiogenesis and support invasion and metastasis. Therefore, serum uric acid level may be a good predictor for metastasis in patients with rectal cancer [30].

In another study, Singh et al. [33] in India compared the salivary antioxidant level of uric acid, glutathione S transferase, and superoxide dismutase between healthy controls and OSCC patients. Also, they compared the study subgroups based on clinical stage and histopathological grade of cancer. They found that the mean salivary levels of uric acid, glutathione S transferase, and superoxide dismutase in OSCC patients were significantly lower compared with healthy controls, and glutathione S transferase and superoxide dismutase levels were not significantly different based on clinical stages; whereas, the uric acid level showed a progressive reduction from stage I to stage IV, although this reduction was not statistically significant.

The current study had some limitations such as small sample size and diet as a confounding factor. The results of this study did not support the role of uric acid as a protective biomarker in different grades of OSCC. No significant relationship was found between the histopathological grade and the serum level of uric acid either. Future multicenter or cohort studies with a larger sample size are required to further elucidate this topic.

## Figures and Tables

**Table 1:** Gender and age of participants, location of OSCC lesions, and histopathological tumor grade.

	Case	Control	*P* value
Gender			
Male	20 (50%)	20 (50%)	1
Female	20 (50%)	20 (50%)	
Age (years)	46.43 ± 10.57	47.3 ± 8.76	0.244
Tumor location	Number (percentage)		
Tongue	20 (50%)		
Buccal mucosa	10 (25%)		
Lower lip	5 (12.5%)		
Floor of the mouth	3 (7.5%)		
Upper lip	2 (5%)		
Total	40 (100%)		
Histopathologic grade	Number (percentage)		
Well differentiated	18 (45%)		
Moderately differentiated	14 (35%)		
Poorly differentiated	8 (20%)		
Undifferentiated	0		
Total	40 (100%)		

**Table 2:** Frequency of histopathological grade according to the location of OSCC.

Location	Grade 1	Grade 2	Grade 3
Tongue	8 (44.4%)	8 (57.1%)	4 (50.0%)
Buccal mucosa	4 (22.2%)	4 (28.6%)	2 (25.0%)
Lower lip	3 (16.7%)	2 (14.3%)	0 (0%)
Upper lip	2 (11.1%)	0 (0%)	0 (0%)
Floor of the mouth	1 (5.6%)	0 (0%)	2 (25.0%)

**Table 3:** Comparison of serum uric acid level between the patient and control groups.

Serum uric acid level (mg/dL)	Groups		*P* value
	Patient	Control	
Mean ± std.deviation	4.2 ± 1.5	4.38 ± 1.22	0.377
Minimum	0.64	3	
Maximum	7.9	8.2	

**Table 4:** Serum uric acid level in the patient group according to gender.

Serum uric acid level	Gender		P-value
	Male	Female	
Mean ± std.deviation (mg/dL)	4.43 ± 1.66	4 ± 1.32	0.201
Minimum	1.8	0.64	
Maximum	7.9	5.8	

**Table 5:** Correlation of histopathological tumor grade and serum uric acid level.

Uric acid level	Grade			*P* value
	Mild	Moderate	High	
Mean ± std.deviation				
(mg/dL)	4 ± 1.36	4.35 ± 1.83	4.36 ± 1.39	*P* = 0.781
Minimum	1.4	0.64	2.1	
Maximum	5.9	7.9	6.9	

## Data Availability

The data used to support the findings of this study are available from the corresponding author upon request.
